# Comparison of the body proximate compositions of juvenile bronze gudgeon (*Coreius heterodon*) and largemouth bronze gudgeon (*C. guichenoti*) in the upstream region of the Yangtze River

**DOI:** 10.1186/2193-1801-2-75

**Published:** 2013-02-28

**Authors:** Yiping Luo, Qingda Huang, Yurong Zhang, Shuting Liu, Wen Wang

**Affiliations:** Key Laboratory of Freshwater Fish Reproduction and Development (Southwest University), Ministry of Education, Chongqing, 400715 China

**Keywords:** Body size, Water content, Lipid content, Energy content, Bronze gudgeon

## Abstract

The body proximate compositions were assessed in juvenile *Coreius heterodon* and *C. guichenoti* from the upstream of the Yangtze River. The migratory *C. guichenoti* has a higher lipid content (*FAT*) than the residential *C. heterodon*. *FAT* of *C. guichenoti* showed an interesting pattern of increase, where *FAT* increased up rapidly and then leveled off as body mass (*M*) increased above 6.5 g, suggesting that the lipid concentration reaches an upper limit of deposition. In both species, *FAT* of the smaller individuals was lower than protein content (*PRO*), but *FAT* increased more rapidly than *PRO* as the fish grew. This indicates that more energy was allocated to protein synthesis than lipid in the smaller fish, with an energy allocation shift from protein synthesis to lipid storage as the fish grew. Strong relationships between both *FAT* and energy content (*E*) and water content (*WAT*) were found in both species, suggesting strong predictive power for future application. However, different models for the two species should be used to predict *FAT* or *E* by *WAT*.

## Introduction

Fish body proximate compositions are important parameters used in fish ecology and physiology, and are related to feeding status ( Sogard and Spencer 2004 ; Blake et al. 2006 ; Ali et al. 2008 ), seasons ( Jonsson et al. 1997 ; Berg and Bremset 1998 ; Robards et al. 1999 ), habitat ( Anthony et al. 2000 ; Dempson et al. 2004 ), and body size ( Deegan 1986 ; Shearer et al. 1994 ; Jonsson and Jonsson 1998 , 2003 ). Changes in the proportions of water, lipid, protein, and ash, result in variation of energy storage in the fish body. This might influence performance related to species fitness, such as the chance of the successful reproduction, survival during seasonal food shortages, and avoidance of predation. In many fish species, the contents of lipid, protein, and energy increase as the fish grows, accompanied by a decrease in water content ( Shearer et al. 1994 ; Jonsson and Jonsson 1998 , 2003 ; Anthony et al. 2000 ). Data for more species needs to be documented.

Previous studies have found strong relationships between water content or dry mass content and other proximate compositions ( Jonsson and Jonsson 1998 , 2003 ; Pangle and Sutton 2005 ; Hartman and Margraf 2008 ). Water content is relatively simple to measure, therefore it can be used as an easy indicator to estimate concentrations of lipid, protein, and energy ( Van Pelt et al. 1997 ; Hartman and Margraf 2008 ). However, prediction models can vary among species ( Hartman and Margraf 2008 ). It is of interest to examine whether a common prediction model is possible for some closely related species.

The bronze gudgeon, *Coreius heterodon*, and the largemouth bronze gudgeon, *Coreius guichenoti*, are two related species of wild freshwater fish in China. The two species have many similar biological characteristics, such as body shape, feeding habits and growth parameters. Both are of economic importance, and are the main species found in the mid- and upstream parts of the Yangtze River, accounting for 34% (*C. heterodon*) and 24% () of total species abundance (Yang et al. *C. guichenoti*^2012^ ). Their population dynamics ( Yang et al. 2012 ), population genetics ( Liao et al. 2007 ; Zhang and Tan 2010 ), reproductive ecology ( Liu et al. 1990 ) and respiratory physiology ( Luo and Wang 2012 ) have been reported in previous studies. *C. heterodon* resides in a limited river area for its whole life, while *C. guichenoti* has a special migratory-like behavior. Juvenile *C. guichenoti* live in the area of Chongqing for around 3 to 4 years, then gradually move 600–1000 km up to the Jinsha River (the upper reaches of Yangtze River above Yibin City) and are resident in Jinsha River for their whole lives ( Liu et al. 1990 ; Ding, 1994 ). Adult fish spawn in the lotic and cooler water from April to July each year and the eggs drift downstream into the Yangtze River ( Liu et al. 1990 ). Long distance movement is an energy expensive process ( Hinch and Bratty 2000 ; Kiessling et al. 2004 ; Caudill et al. 2007 ), therefore it could be hypothesized that the fish store sufficient energy before starting to move upstream. However, energy accumulation and growth in the juveniles of this fish are unclear. Therefore, it is of interest to study how the body chemical compositions of juveniles of this species change as their body mass increases. Previous studies have shown that long distance migratory species have greater energy storage and faster deposition of lipids with body growth ( Jonsson and Jonsson 1998 ; Jonsson and Jonsson 2005 ). Comparison of the body chemical compositions of the migratory *C. guichenoti* and the residential *C. heterodon* could provide new data for closely related species with different life-history strategies. We hypothesize that *C. guichenoti* has a higher body lipid content of *C. heterodon*.

This study aims to provide energy prediction models for juvenile *C. guichenoti* and *C. heterodon* in the upstream part of the Yangtze River and to determine how the proximate compositions change with body growth in the closely related species with different life-histories.

## Materials and methods

The fish used in this study were collected from the Chongqing area (106°50^′^E, 29°35^′^N) of the upstream part of the Yangtze River in early September 2010. For a map of the sample region, refer to Luo and Wang ( 2012 ). Large square nets (1.5 cm×1.5 cm) and gill nets (10 cm×10 cm) were used to sample fish. Wet mass (*M*, g) of the whole fish was measured to 0.01 g accuracy and body length (*L*, cm) was measured to 0.01 cm. The fish were sealed in polyethylene bags and frozen immediately. Fish bodies were dried at 70°C for 5 to 15 days until constant mass was obtained to determine the dry mass (g) and water mass (g). The water content (*WAT*,%) was calculated from the percentage of water mass to wet body mass. Protein content (*PRO*,%) was determined by the Kjeldahl method. Lipid content (*FAT*,%) was determined by ether extraction using Soxhlet. Ash content (*ASH*,%) was determined by combustion at 550°C for 7 hours. Energy content (*E*, kJ g^-1^) was calculated based on 23.6 kJ g^-1^ of protein and 39.5 kJ g^-1^ of lipid ( Brett and Groves 1979 ). Small individuals of similar size (body length difference within 2 mm) were pooled to obtain sufficiently large samples for chemical analysis (15 g wet mass). The mean value of the body length, the body mass, and the chemical composition of each pool was used as one sample. The final sample sizes were 53 for *C. heterodon* and 118 for *C. guichenoti*.

We used SPSS 11.0 (SPSS Inc., Chicago, IL, USA) for statistical analyses. The relationships between *M* and chemical composition were described using power curve estimation. The relationships between *WAT* and other proximate compositions were described using linear regression. General linear model (GLM) followed by least significant difference tests were used to compare the slopes or power exponents and intercepts between the two species, with *M* as a covariate. Differences were considered significant when the *P*-value was less than 0.05.

## Results

**Figure 1:**
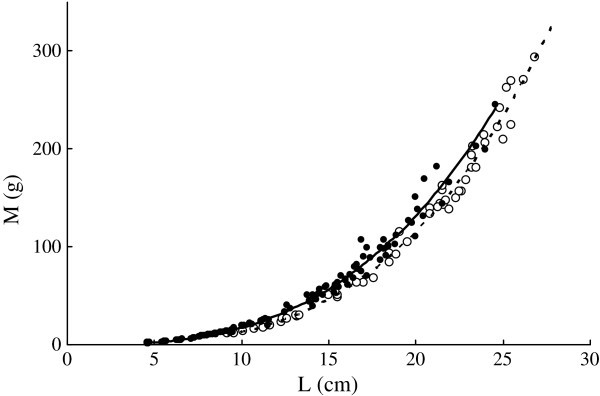
**Relationships between body length (*L*, cm) and wet body mass (*M*, g) of*C. heterodon*(*M*** **= 0.0086*L***^**3.17**^**,*r***^**2**^ **= 0.995,*n*** **= 53,*P*** **< 0.01) and*C. guichenoti*(*M*** **= 0.0205*L***^**2.93**^**,*r***^**2**^ **= 0.996,*n*** **= 118,*P*** **< 0.01)*.*** The empty circle and dotted curve indicate *C. heterodon*; the solid circles and curve indicate *C. guichenoti*.

*L* of *C. heterodon* ranged from 9.2 to 26.8 cm with *M* varying from 11.3 to 293.2 g. *L* of *C. guichenoti* ranged from 4.6 to 24.6 cm with *M* varying from 1.5 to 245.7 g. *M* and *L* were significantly correlated in both species (Figure 1).

**Figure 2:**
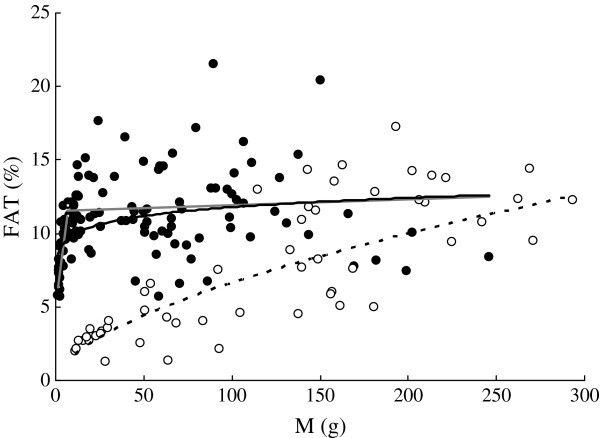
**Relationships between wet body mass( *M* , g)and body lipid content (*FAT*,%) of*C. heterodon*(*FAT*** **= 0.436*M***^**0.591**^**,*r***^**2**^ **= 0.651,*n*** **= 53,*P*** **< 0.01) and*C. guichenoti*(*FAT*** **= 8.33*M***^**0.0753**^**,*r***^**2**^ **= 0.152,*n*** **= 118,*P*** **< 0.01)*.*** The empty circle and dotted curve indicate *C. heterodon*; the solid circles and curve indicate *C. guichenoti*. The grey lines indicate the two-line regressions between *FAT* and *M* of *C. guichenoti*. For the fish smaller than transition point (6.5 g), the regression was *FAT* = 5.49 + 0.872*M* (*r*^2^ = 0.903, *n* = 24, *P* < 0.0001), while for the bigger fish, the regression was *FAT* = 11.8 + 0.00290*M* (*r*^2^ = 0.177, *n* = 94, *P* > 0.05).

*FAT* ranged from 2.02% to 14.6% in *C. heterodon* and from 5.66% to 20.4% in *C. guichenoti* (Figure 2). *FAT* increased with *M* in both *C. heterodon* and *C. guichenoti*. The regression of *FAT* (%) and *M* (g) was *FAT* = 0.436*M*^0.591^ (*r*^2^ = 0.651, *n* = 53, *P* < 0.01) for *C. heterodon* and was *FAT* = 8.33*M*^0.0753^ (*r*^2^ = 0.152, *n* = 118, *P* < 0.01) for *C. guichenoti*. *FAT* of *C. guichenoti* was significantly higher than that of *C. heterodon* using GLM with *M* as a covariate (F_1, 170_ = 24.19, *P* < 0.01). Since *FAT* of *C. guichenoti* increased and then leveled off as *M* increased, two-line regressions were also used to describe the relationship between *FAT* and *M* for this species. The transition point of these regressions was at *M* = 6.5 g. For the fish smaller than transition point, the regression was *FAT* = 5.49 + 0.872*M* (*r*^2^ = 0.903, *n* = 24, *P* < 0.0001), while for the bigger fish, the regression was *FAT* = 11.8 + 0.00290*M* (*r*^2^ = 0.177, *n* = 94, *P* > 0.05).

**Figure 3:**
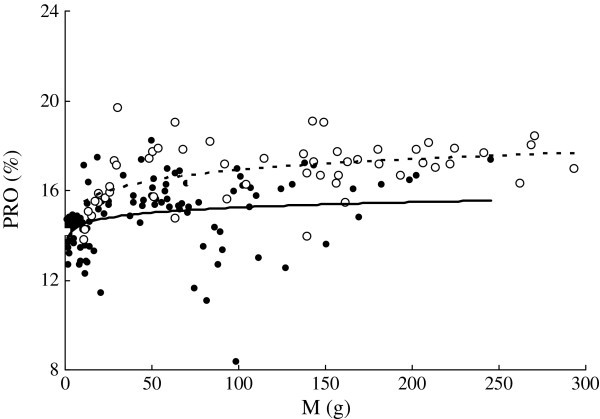
**Relationships between wet body mass (*M*, g) and body protein content (*PRO*,%) of*C. heterodon*(*PRO*** **= 14.0*M***^**0.0410**^**,*r***^**2**^ **= 0.234,*n*** **= 53,*P*** **< 0.01) and*C. guichenoti*(*PRO*** **= 13.8*M***^**0.0221**^**,*r***^**2**^ **= 0.074,*n*** **= 118,*P*** **< 0.01)*.*** The empty circle and dotted curve indicate *C. heterodon*; the solid circles and curve indicate *C. guichenoti*.

*PRO* ranged from 13.8% to 19.1% in *C. heterodon* and from 8.40% to 17.4% in *C. guichenoti* (Figure 3). *PRO* (%) of both species were also significantly correlated with *M* (Figure 3). No significant difference was found in exponent (*b*) values in the regressions between the two species, using *M* as a covariate (F_1, 170_ = 0.515, P > 0.05). *PRO* of *C. heterodon* was significantly higher than that of *C. guichenoti,* using *M* as a covariate.

**Figure 4:**
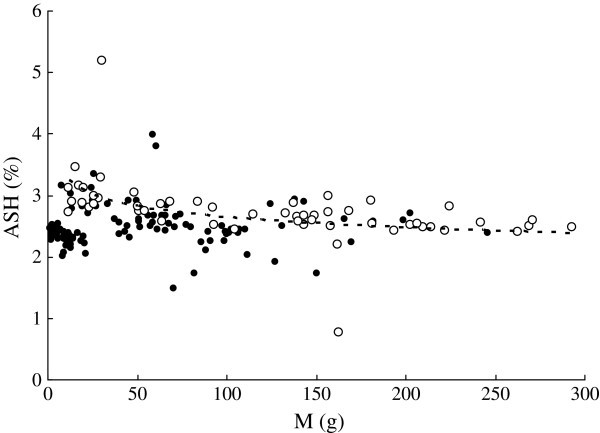
**Relationships between wet body mass (*M*, g) and ash content (*ASH*,%) of*C. heterodon*(*ASH*** **= 4.11*M***^**-0.949**^**,*r***^**2**^ **= 0.166,*n*** **= 53,*P*** **< 0.01) and*C. guichenoti.*** The empty circle and dotted curve indicate *C. heterodon*; the solid circles *C. guichenoti*.

*ASH* ranged from 0.78% to 5.20% in *C. heterodon* and from 1.49% to 3.15% in *C. guichenoti* (Figure 4). *ASH* (%) of *C. heterodon* was significantly correlated with *M* (Figure 4). No significant correlation was found between *ASH* and *M* of *C. guichenoti*. *ASH* of *C. heterodon* was significantly higher than that of *C. guichenoti* using GLM with *M* as a covariate (F_1, 170_ = 11.71, *P* < 0.01).

**Figure 5:**
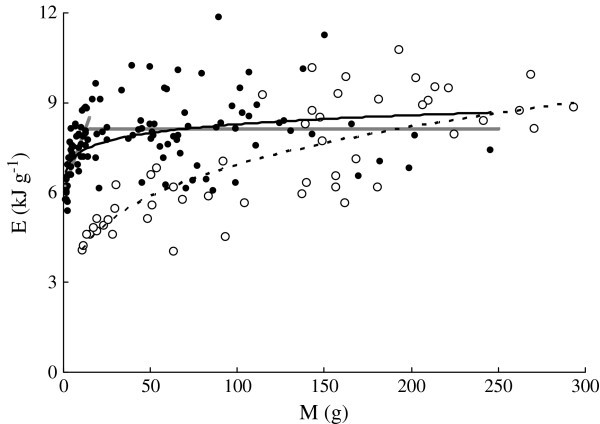
**Relationships between wet body mass (*M*, g) and energy content (*E*kJ g**^**-1**^**) of*C. heterodon*(*E*** **= 2.27*M***^**0.242**^**,*r***^**2**^ **= 0.667,*n*** **= 53,*P*** **< 0.01) and*C. guichenoti*(*E*** **= 6.55*M***^**0.0511**^**,*r***^**2**^ **= 0.219,*n*** **= 118,*P*** **< 0.01).** The empty circle and dotted curve indicate *C. heterodon*; the solid circles and curve indicate *C. guichenoti*. The grey lines indicate the two-line regressions between *E* and *M* of *C. guichenoti*. For the fish smaller than transition point (19.1 g), the regression was *E* = 6.12 + 0.165*M* (*r*^2^ = 0.705, *n* = 50, *P* < 0.0001), while for the bigger fish, the regression was *E* = 8.13 + 0.00000809*M* (*r*^2^ = 0.508, *n* = 68, *P* > 0.05).

*E* ranged from 4.02 to 10.8 kJ g^-1^ in *C. heterodon* and from 5.37 to 11.3 kJ g^-1^ in *C. guichenoti* (Figure 5). *E* (kJ g^-1^) of both species were significantly correlated with *M* (Figure 5). *E* of *C. guichenoti* was significantly higher than that of *C. heterodon* using GLM with *M* as a covariate (F_1, 170_ = 19.72, *P* < 0.01). For the fish smaller than the transition point (19.1 g), the regression was *E* = 6.12 + 0.165*M* (*r*^2^ = 0.705, *n* = 50, *P* < 0.0001), while for the bigger fish, the regression was *E* = 8.13 + 0.00000809*M* (*r*^2^ = 0.508, *n* = 68, *P* > 0.05).

**Figure 6:**
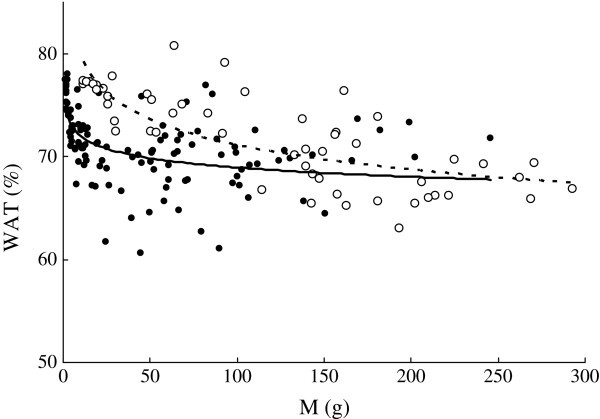
**Relationships between wet body mass (*M*, g) and water content (*WAT*,%) of*C. heterodon*(*WAT*** **= 89.2*M***^**-0.0492**^**,*r***^**2**^ **= 0.555,*n*** **= 53,*P*** **< 0.01) and*C. guichenoti*(*WAT*** **= 75.2*M***^**-0.0189**^**,*r***^**2**^ **= 0.262,*n*** **= 118,*P*** **< 0.01).** The empty circle and dotted curve indicate *C. heterodon*; the solid circles and curve indicate *C. guichenoti*.

*WAT* (%) ranged from 63.0% to 80.7% in *C. heterodon* and from 60.6% to 78.0% in *C. guichenoti* and both decreased with *M* (g) (Figure 6). The *b*-value of the regression was significantly lower (F_1, 170_ = 8.04, *P* < 0.01), while the intercept was significantly higher in *C. heterodon* (F_1, 170_ = 26.08, *P* < 0.01) than those of *C. guichenoti,* using GLM with *M* as a covariate.

**Figure 7:**
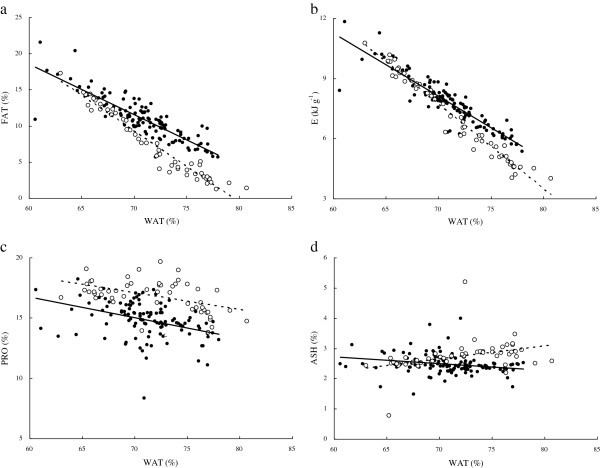
**Relationships between body water content (*WAT*,%) and other proximate compositions of*C. heterodon*and*C. guichenoti*. a**: *FAT* = 60.3-0.695*WAT*, *r*^2^ = 0.731, *n* = 53, *P* < 0.01 for *C. heterodon*; *FAT* = 78.2-0.984*WAT*, *r*^2^ = 0.950, *n* = 118, *P* < 0.01 for *C. guichenoti*. **b**: *E* = 30.1-0.314*WAT*, *r*^2^ = 0.824, *n* = 53, *P* < 0.01 for *C. heterodon*; *E* = 37.3-0.422*WAT*, *r*^2^ = 0.964, *n* = 118, *P* < 0.01 for *C. guichenoti*. **c**: *PRO* = 27.0-0.171*WAT*, *r*^2^ = 0.153, *n* = 53, *P* < 0.01 for *C. heterodon*; *PRO* = 27.0-0.141*WAT*, *r*^2^ = 0.237, *n* = 118, *P* < 0.01 for *C. guichenoti*. **d**: *ASH* = 4.03-0.022*WAT*, *r*^2^ = 0.054, *n* = 53, *P* < 0.05 for *C. heterodon*; *ASH* = −0.338 + 0.0429*WAT*, *r*^2^ = 0.150, *n* = 118, *P* < 0.05 for *C. guichenoti*. The empty circle and dotted curve indicate *C. heterodon*; the solid circles and line indicate *C. guichenoti*.

*WAT* was negatively linearly correlated with *FAT* or *E* in both species (Figure 7 a, b). A significant difference was found in the regression slopes between the two species (ANCOVA, F_1, 170_ = 26.94 for *FAT*, *P* < 0.01; F_1, 170_ = 31.49 for E, *P* < 0.01). Significant correlations were also found between *WAT* and both *PRO* and *ASH* but with smaller *r*^2^ values (Figure 7 c, d).

## Discussion

The exponent values of the length-mass relationships of both *C. heterodon* (3.17) and *C. guichenoti* (2.93) estimated in this study (Figure 1) were within the general range found for fishes (2.5 to 3.5) in previous studies ( Carlander 1969 ). The exponent value of *C. heterodon* indicates a positively allometric growth, while the exponent value of *C. guichenoti* (2.93) indicates an approximately isometric growth. The faster increase of weight than length of *C. heterodon* in this study indicates that bone mass tends to increase at a slower rate than does muscle mass, as stated by Shearer ( 1994 ). Correspondingly, in this study *ASH* decreased with *M* of *C. heterodon* (Figure 4), which could reflect a slower increase in bone mass. The isometric growth of *C. guichenoti* is also consistent with its unchanged *ASH* with *M*.

*FAT* of *C. guichenoti* was relatively higher than that of *C. heterodon* (Figure 2). Childs and King ( 1993 ) classified fishes into low fat fish (*FAT* within 0.6-3.0%), intermediate fat fish (*FAT* within 3.5% to 7.0%) and high fat fish (*FAT* within 8.5% to 15.3%). *C. guichenoti* (*FAT* ranges 5.66% to 20.4%) could be incorporated into the intermediate or high fat categories and some individuals of larger size even having *FAT* above the range of high fat categories. *C. heterodon* (*FAT* ranges 2.02% to 14.6%) could be incorporated into intermediate or low fat categories. It has been found that the long distance migratory species deposit larger amounts of body lipid ( Jonsson and Jonsson 2005 ), which could be the reason for the higher *FAT* of the migratory *C. guichenoti* than that of the residential *C. heterodon*.

*FAT*, *PRO*, and *E* of many fish species increase as the body size increases ( Berg and Bremset 1998 ; Jonsson and Jonsson 1998 , 2003 ; Sogard and Spencer 2004 ). Similar results were also found in the both species in this study (Figures 2, 3 and 5). However, *FAT* of *C. guichenoti* showed an interesting pattern of increase, where *FAT* increased rapidly and then leveled off as *M* increased above 6.5 g. *E* of *C. guichenoti* also increased in a similar two-line pattern but with a relative larger transition body mass of 19.1 g. This was partly contributed to by the persistent increase in *PRO*. This suggests that the lipid concentration of *C. guichenoti* reaches an upper limit. Thus, the energy storage of larger fish needed for migration may depend mainly on increase of body size rather than body energy density.

*PRO* of the smaller individuals of both species was higher than *FAT*, indicating that more intake energy was allocated to synthesis of protein than lipid (Figures 2 and 3). Similar results were also reported in Atlantic salmon (*Salmo salar*), brown trout (*S. trutta*), and sablefish () (Berg and Bremset *Anoplopoma fimbria*^1998^ ; Jonsson and Jonsson 1998 ; Sogard and Spencer 2004 ). The strategy of protein build-up of fish fry might enhance their competition capacity and reduce predation risk ( Calow 1985 ). Furthermore, *FAT* of both species increases faster than *PRO* as the fishes grow. This suggests that the importance of lipid increases and there is a shift in energy allocation from protein synthesis to lipid storage. Similar results were also found in gulf menhaden () (Deegan *Brevoortia patronus*^1986^ ) and many other species ( Shearer et al. 1994 ; Anthony et al. 2000 ).

Previous studies ( Hartman and Brandt, 1995 ; Jonsson and Jonsson 1998 ; Pangle and Sutton 2005 ; Hartman and Margraf 2008 ) have suggested using dry mass content or water content to predict the proximate composition of fishes. The present study also found significant relationships between *WAT* and *FAT*, *PRO*, *ASH*, or *E* in both fish species (Figure 7 a, b, c, d). The models for *FAT* or *E* of both species yielded high *r*^2^ values (range: 0.731 to 0.964) suggesting strong predictive power for future application. However, variation of *PRO* and *ASH* could be explained less well by *WAT* (*r*^2^ values range: 0.054 to 0.237). The lower predictive power of models for *PRO* and *ASH* may be related to narrow ranges of protein and ash contents in both *C. heterodon* and *C. guichenoti*. Fishes may exchange body water and fat when energy budgets change ( Hartman and Margraf 2008 ). Previous studies suggest that the equal amounts of decrease in body water are associated with the accumulation of around three times as much lipid as protein ( Schmidt-Nielsen 1975 ; Jobling 1994 ). The present results showed that the slopes of *WAT*-*FAT* model were 4.1 times higher in *C. heterodon* and 2.3 times higher in *C. guichenoti,* compared with the slopes of *WAT*-*PRO* model, suggesting a rapider accumulation of lipid. Similar rapider accumulation of lipid was also found in brown trout ( Jonsson and Jonsson 1998 ) and Atlantic salmon ( Jonsson and Jonsson 2003 ).

Our results also showed significant differences between the two species of the slopes in both *WAT*-*FAT* and *WAT*-*E* models (Figure 7 a, b). Equal changes in body water would induce 1.4 times the change in *FAT* and 1.3 times the change in *E* for *C. guichenoti* compared with *C. heterodon*. This suggests that, even between closely related species, *FAT* and *E* cannot be predicted by *WAT* using general models. Further work is needed to determine whether the stronger replacement between water and lipid in *C. guichenoti* is related to its migratory characteristics.

For future work, different models for the two species should be used to predict *FAT* or *E* by *WAT*. The migratory *C. guichenoti* has a higher *FAT* than that of the residential *C. heterodon*. With dam constructions in the upstream region of the Yangtze River, *C. guichenoti* is undergoing loss of its migratory pathway and even its migratory behavior. Its energetic response to the intense changes of habitat remains unclear and would be an interesting area of future research.
